# QTL mapping reveals the genetic architecture of loci affecting pre- and post-zygotic isolating barriers in Louisiana Iris

**DOI:** 10.1186/1471-2229-12-91

**Published:** 2012-06-15

**Authors:** Evangeline S Ballerini, Amanda N Brothers, Shunxue Tang, Steven J Knapp, Amy Bouck, Sunni J Taylor, Michael L Arnold, Noland H Martin

**Affiliations:** 1 Department of Genetics, University of Georgia, Athens, GA, USA; 2 Dow AgroSciences LLC, Indianapolis, IN, USA; 3 Monsanto, Woodland, CA, USA; 4 Illumina, Inc., San Diego, CA, USA; 5 Department of Biology, Texas State University, San Marcos, TX, USA

## Abstract

**Background:**

Hybridization among Louisiana Irises has been well established and the genetic architecture of reproductive isolation is known to affect the potential for and the directionality of introgression between taxa. Here we use co-dominant markers to identify regions where QTL are located both within and between backcross maps to compare the genetic architecture of reproductive isolation and fitness traits across treatments and years.

**Results:**

QTL mapping was used to elucidate the genetic architecture of reproductive isolation between *Iris fulva* and *Iris brevicaulis*. Homologous co-dominant EST-SSR markers scored in two backcross populations between *I. fulva* and *I. brevicaulis* were used to generate genetic linkage maps. These were used as the framework for mapping QTL associated with variation in 11 phenotypic traits likely responsible for reproductive isolation and fitness. QTL were dispersed throughout the genome, with the exception of one region of a single linkage group (LG) where QTL for flowering time, sterility, and fruit production clustered. In most cases, homologous QTL were not identified in both backcross populations, however, homologous QTL for flowering time, number of growth points per rhizome, number of nodes per inflorescence, and number of flowers per node were identified on several linkage groups.

**Conclusions:**

Two different traits affecting reproductive isolation, flowering time and sterility, exhibit different genetic architectures, with numerous QTL across the *Iris* genome controlling flowering time and fewer, less distributed QTL affecting sterility. QTL for traits affecting fitness are largely distributed across the genome with occasional overlap, especially on LG 4, where several QTL increasing fitness and decreasing sterility cluster. Given the distribution and effect direction of QTL affecting reproductive isolation and fitness, we have predicted genomic regions where introgression may be more likely to occur (those regions associated with an increase in fitness and unlinked to loci controlling reproductive isolation) and those that are less likely to exhibit introgression (those regions linked to traits decreasing fitness and reproductive isolation).

## Background

Hybridization between species is a relatively common phenomenon that has been well documented in both animals [1] and plants [2] and may play an important role in the process of speciation. In plants, hybridization has been hypothesized to be especially extensive, and it has recently been shown that in plant families with two or more species, nearly half of those surveyed were found to have hybrids (48.5 %, [2]). Ultimately, the evolutionary outcomes of natural hybridization will depend on the nature of the reproductive barriers that act to reduce gene flow between the hybridizing species pairs; the rate of F_1_ hybrid formation will be directly affected by the number (and strength) of pre-zygotic isolating barriers, while post-zygotic isolating barriers will act directly on F_1_ and later-generation hybrids to further reduce the likelihood of gene flow. Normally, a combination of pre- and post-zygotic isolating barriers act in concert to affect the total isolation observed between taxa [3,4]. However, even in cases where the total isolation measured is near-complete (e.g. F_1_ hybridization is rare and F_1_ and later generation hybrids are relatively unfit), interspecific gene flow is still possible, as demonstrated by studies documenting the ability of genomic regions to cross species boundaries [5-10]. The potential for introgression of genomic regions influencing quantitative variation between species is dependent on the underlying genetic architecture (such as the number of loci, the magnitude and effect of each locus, and interactions between loci) of the diverse isolating barriers preventing gene flow between the hybridizing (even rarely hybridizing) species [11].

Quantitative trait locus (QTL) mapping studies are an effective tool for visualizing the genetic architecture of traits important for reproductive isolation, fitness, and survival [9,12-16]. Following the genic view of speciation, differences in a relatively small number of loci are sufficient to establish isolation between species [17]). While these loci (‘speciation genes’) are not free to move across species boundaries due to negative fitness effects when placed in a heterologous genetic background, the portions of the genome that are not linked to these loci could potentially be tolerant to introgression [17,18]. Therefore, by examining the direction of QTL effects for traits influencing reproductive isolation and fitness, QTL mapping studies can be used to predict both regions of the genome that are resistant to introgression (potentially identifying regions tightly linked to ‘speciation genes’), as well as genomic regions that are more likely to introgress (regions of the genome that introgress with neutral or positive effects on fitness).

The potential for introgression (or lack thereof) of any particular genomic region will be influenced by the presence of QTL affecting reproductive isolation and hybrid fitness, and the direction of the effects that those QTL have on fitness. For example, a genomic region tightly linked to a QTL where introgression of the heterospecific allele results in sterility would likely not introgress across species boundaries. To date, the majority of QTL studies examining genetic architecture focus on single traits, however, multiple barriers are usually responsible for affecting reproductive isolation between hybridizing species pairs [3]. Consequently, accurately predicting the presence and direction of gene flow at any particular locus will require determining whether or not that locus is linked to QTL that affect reproductive isolation and hybrid fitness.

In this study, we use a previously-published linkage map based on co-dominant microsatellite markers to map various phenological, morphological, and ecological traits affecting reproductive isolation and fitness for two species of the well-studied Louisiana Iris system, *Iris brevicaulis* and *Iris fulva*. These species occur throughout southern Louisiana and can be found along the Mississippi River drainage basin as far north as the Great Lakes region ([19]; http://www.PLANTS.usda.gov). They are relatively long-lived perennials that have the ability to reproduce asexually by vegetative propagation of rhizomatous stems. Previous studies have identified several traits that contribute to the maintenance of species boundaries between *I. brevicaulis* and *I. fulva*, including the partitioning of habitat space via water-level preference and tolerance [9,15,19,20], shifts in flowering phenology [4,19,21], and differences in floral morphology and pollinator visitation [22,23]. For example, *I. fulva* commonly grows along bayou edges and produces crimson-colored flowers primarily visited by hummingbirds and butterflies in late March through early May. In contrast, *I. brevicaulis* is found in drier shadier habitats and produces blue flowers with yellow nectar guides primarily visited by bees in late April through early June. However, when found in sympatry in southern Louisiana, natural hybrid zones form (e.g. [19,24]). F_1_ hybrids are both viable and fertile, often exhibiting heterosis [25-27], and through backcrossing to parental species, can produce later generation hybrids that may facilitate the introgression of alleles between species. The genetic basis of the traits affecting reproductive isolation and the fitness of hybrid offspring has been previously examined by QTL mapping using two separate unlinked genetic maps using reciprocal first generation backcross mapping populations [4,9,15,22,23,26]. We compiled phenotypic data from multiple traits that potentially affect prezygotic isolation and hybrid fitness in *I. fulva* X *I. brevicaulis* hybrids and utilized new high density genetic maps created with co-dominant markers to 1) identify QTL affecting the life history, fitness, and reproductive isolation of *I. fulva* and *I. brevicaulis*; 2) detect regions where QTL are clustered on certain linkage groups such that recombination among the traits is likely to be limited; and 3) compare data across generation, year, and treatment on a single QTL map. In addition, using co-dominant markers will allow us to use the same markers to screen natural hybrid zones to determine whether or not these QTL exhibit directional introgression in nature.

## Results

**Figure 1:**
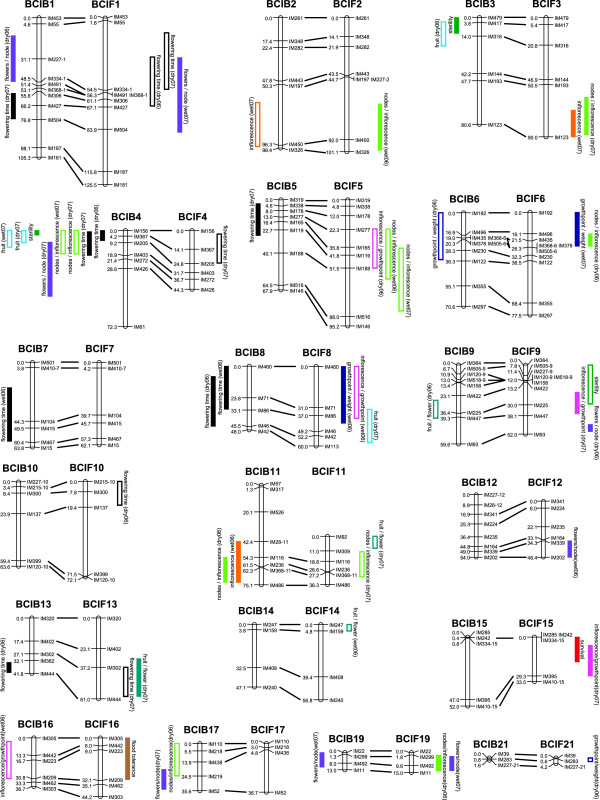
**Locations of QTL on homolgous BCIB and BCIF linkage groups. **The 2-LOD confidence interval for each QTL is presented. Outlined bars indicate QTL with positive additive effects, solid bars indicate QTL with negative additive effects. Traits are color coded as follows: black – flowering time; flood tolerance – brown; long-term survival – red; sterility – dark green; growth points/weight – dark blue; inflorescence production – orange; proportion of growth points producing inflorescences – pink; nodes per inflorescence – light green; flowers per node – purple; fruit production – light blue; proportion of flowers that produce fruits - turquoise. Linkage groups 12, 18, and 20 do not have any QTL and are not shown.

Across all traits examined, we identified a total of 25 QTL in the BCIB and 31 QTL in the BCIF population. It is likely that additional QTL may exist for the traits presented here, but they were not detected because the effect is too small to be detected with the sample sizes used in these analyses. This may be especially true for traits dependent on having plants that flowered (i.e. flowering phenology, traits related to the inflorescence), as phenotypes could only be assayed in a limited number of individuals for these traits. Moreover, the reduced sample size may inflate the effect size of the QTL, consequently we focus on the presence or absence of QTL and do not focus on their effect size [28]. The positions of QTL are distributed across much of the genome with 12 of the 21 linkage groups having QTL detected in both mapping populations, two linkage groups have QTL that were detected only in the BCIB map, while five linkage groups have QTL that were detected only in the BCIF map (Figure1). Linkage groups (LGs) 18 and 20 had no QTL detected in either of the mapping populations (not displayed in Figure1). Several different traits revealed QTL that colocalized in the same locations, most notably in the BCIB map where QTL for five traits can be found to colocalize on LG 4. Interestingly, a QTL for only one of these traits is found on the reciprocal LG 4 in the BCIF map. 

In previous QTL mapping studies performed on much of these data, we were unable to determine whether QTL identified on one linkage map corresponded to QTL identified on the reciprocal map. Because of our codominant marker system, we were able to identify traits with QTL located in homologous chromosomal regions between the BCIB and BCIF maps (Table 1; Figure1). In few cases did we recover collinear QTL for the same trait in each backcross direction, a phenomenon that can be explained in part by the inability to determine the effects of dominant QTL in backcross designs, however we were able to identify collinear QTL for four traits.

**Table 1:** Summary of co-localized QTL

**Trait**	***I. brevicaulis *alleles in BCIF**	***I. fulva *alleles in BCIB**	**QTL overlapping on both maps**
**Flowering time**	4 QTL (two overlapping on LG1), all increase the time to flowering	9 QTL (2 sets overlapping, LG4 and LG8), all decrease time to flowering	Overlapping with consistent effects on LG4 and LG13
**Flood tolerance**	1 QTL, decreases survival	No QTL	None
**Long-term survival**	1 QTL, decreases survival	No QTL	None
**Sterility**	1 QTL that increases sterility	2 QTL that decrease sterility	None
**Growth points/weight**	3 QTL, 2 decrease, 1 increases the growth points/weight	1 QTL, increases the growth points/weight	Overlapping with consistent effects on LG6
**Inflorescence production**	1 QTL, increases inflorescence production	2 QTL, 1 increases, 1 decreases likelihood of inflorescence production	None
**Proportion growth points producing inflorescences**	4 QTL, 2 increase, 2 decrease ratio of inflorescences per growth point	1 QTL, increases number of inflorescences per growth point	None
**Nodes per inflorescence**	7 QTL, (2 overlap on LG4) 4 decrease, 3 increase nodes per inflorescence	4 QTL, 3 increase (2 overlap) the nodes per inflorescence, 1 decreases nodes per inflorescence	Overlapping with consistent effects on LG11
**Flowers per node**	4 QTL, all decrease number of flowers per node	4 QTL, all decrease number of flowers per node	Overlapping on LG1 and LG19, all decrease the number of flowers per node
**Fruit production**	1 QTL, increases fruit production	2 QTL (overlapping on LG4), both increase fruit production	None
**Proportion of flowers that produce fruits**	3 QTL, 2 increase, 1 decreases the proportion of flowers that produce fruit	1 QTL, increases the proportion of flowers that produce fruits	None

Two homologous flowering time QTL were found on LG 4 and LG 13. Similar to previous flowering phenology studies in this system, *I. fulva* alleles were consistently associated with earlier flowering times, while *I. brevicaulis* alleles were consistently associated with later flowering times. Of all of the flowering phenology QTL identified in this study, most (11 out of 13) were identified in plants from the dry site.

In BCIB, one QTL associated with an increase in the number of growth points produced when *I. fulva* alleles were present was identified on LG 6. This QTL overlaps with a QTL in BCIF that is similarly associated with a decrease in the trait value when *I. brevicaulis* alleles are present. Two additional QTL were identified for growth points in BCIF, one in which *I. brevicaulis* alleles increase the trait value and one in which they decrease it (Table 2). Both the number of floral nodes per inflorescence and the number of flowers per node are associated with several QTL in the BCIB and BCIF backcross populations. Four QTL identified in the BCIB population and seven QTL identified in the BCIF population were associated with number of nodes produced per inflorescence. For the QTL identified, both *I. fulva* alleles and *I. brevicaulis* alleles have mixed effects in the heterospecific background. For example, at three loci, *I. fulva* alleles increase nodes per infloresence in BCIB plants and at one locus *I. fulva* alleles decrease nodes per infloresence (at three loci, *I. brevicaulis* alleles increase the trait, at four loci *I. brevicaulis* alleles decrease the trait; Table 2). The two QTL that overlap in homologous regions of LG 11 are determined by *I. fulva* and *I. brevicaulis* alleles that have consistent effects, with the *I. fulva* allele decreasing nodes per inflorescence and the *I. brevicaulis* allele increasing nodes per inflorescence (Tables 2 and 1). Four QTL in both the BCIB and BCIF maps were identified that affect the number of flowers produced per node. Interestingly, all eight QTL found for this trait are associated with the heterospecific allele decreasing in the trait value regardless of the genetic background, including the two pairs that overlap on LGs 1 and 19.

**Table 2:** QTL results

**Trait**	**LG**	**Position**	**LR**	**Additive Effect**	**R**^**2**^	**2 LOD Interval**
**(a) BCIB QTL**						
Flowering time (days)						
dry 2007, n = 112	1	64.6	32.85	−3.20	0.15	57.1-76.3
dry 2006, n = 115	4	0.0	25.48	−4.71	0.15	0-7.5
dry 2007, n = 112	4	11.2	35.16	−3.22	0.16	0.8-18.7
dry 2007, n = 112	5	13.0	13.2	−1.80	0.05	2.9-26.9
wet 2006, n = 112	7	44.3	13.9	−3.20	0.09	19.1-49.5
wet 2006, n = 112	8	15.0	15.72	−3.76	0.13	0-32.4
dry 2006, n = 115	8	30.8	14.95	−3.74	0.09	7.7-45.3
dry 2006, n = 115	13	40.1	15.22	−4.18	0.10	33.6-41.1
Flood tolerance^1^ (n = 145)	No QTL detected	-	-	-	-	-
Long-term survival^1^ (n = 139)	No QTL detected	-	-	-	-	-
Pollen sterility^2^ (n = 184)	3	0.0	14.61	−17.42	0.05	0-11.9
	4	0.0	63.01	−38.26	0.26	0-3.4
Growth points/weight (g)						
dry 2006, n = 158	6	19.0	15.51	0.03	0.08	0-35.3
Inflorescence production^1^						
wet 2007, n = 157	2	78.3	22.24	0.39	0.26	64-97.3
wet 2006, n = 158	11	61.3	12.44	−0.22	0.07	42.4-73.3
Proportion growth points producing an inflorescence						
wet 2006, n = 87	16	13.3	13.31	0.14	0.11	2.7-30.7
Flowering nodes per inflorescence						
dry 2007, n = 87	4	8.2	23.29	0.55	0.19	0-17.6
wet 2007, n = 130	4	9.2	17.94	0.51	0.11	0-17.4
dry 2006, n = 91	11	61.5	15.27	−0.56	0.12	54.3-73.3
dry 2006, n = 91	17	0.0	15.14	0.53	0.11	0-5.5
Flowers per node						
dry 2006, n = 91	1	35.1	14.13	−0.14	0.14	13.6-48.1
dry 2007, n = 87	4	16.2	20.9	−0.09	0.19	8.8-49.6
dry 2007, n = 87	17	24.5	13.7	−0.09	0.11	19.6-34.5
wet 2007, n = 130	19	9.0	17	−0.10	0.11	0-11
Fruit set^1^						
dry 2007, n = 86	4	0.0	47.46	0.51	0.39	0-9.2
wet 2007, n = 130	4	0.0	24.1	0.32	0.14	0-12.5
Proportion of flowers that set fruit						
dry 2006, n = 48	9	37.4	15.15	0.35	0.20	26.7-40.1
**(b) BCIF QTL**						
Flowering time (days)						
dry 2007, n = 104	1	31.8	20.87	3.05	0.30	11.2-53.8
dry 2006, n = 97	1	54.5	21.44	4.67	0.15	29.4-66.4
dry 2007, n = 104	4	19.1	16.04	1.93	0.11	2.6-24.5
dry 2006, n = 107	10	7.8	14.28	3.67	0.09	0-17.9
dry 2007, n = 104	13	57.2	13.80	1.78	0.10	37.2-59.2
Flood tolerance^1^ (n = 145)	16	9	11.27	−0.14	0.07	0-31.4
Long-term survival^1^ (n = 139)	15	0	19.44	−0.22	0.11	0-18.7
Pollen sterility^2^ (n = 116)	9	11.4	11.55	6.85	0.08	0-29.2
Growth points/weight (g)						
wet 2007, n = 69	6	16.1	13.99	−0.12	0.14	0-25.5
wet 2006, n = 68	8	13	13.16	−0.07	0.18	0-42.8
dry 2006, n = 69	21	0	13.33	0.04	0.14	0-2.6
Inflorescence production^1^						
wet 2007, n = 69	3	89.5	13.83	−0.24	0.14	69.9-89.5
Proportion growth points producing an inflorescence						
dry 2006, n = 41	5	22.3	13.58	0.31	0.20	22-51.5
wet 2006, n = 48	8	36	14.27	0.24	0.21	0-39.1
dry 2007, n = 42	9	30	29.00	−0.10	0.34	20.4-36.7
dry 2007, n = 42	15	25	14.07	−0.08	0.17	6.2-29.3
Flowering nodes per inflorescence						
wet 2006, n = 48	2	97	13.54	−0.56	0.15	65.1-100
dry 2007, n = 42	3	80.5	17.75	−0.73	0.30	60.2-88.5
wet 2006, n = 48	5	41.8	16.46	0.59	0.18	22.1-59.8
wet 2007, n = 64	5	54.5	13.65	0.41	0.17	36.5-83.8
dry 2006, n = 41	6	21.5	19.24	−0.78	0.22	16.2-25.6
dry 2007, n = 42	11	18	23.38	1.07	0.30	11.6-30.4
dry 2006, n = 41	19	9.6	19.19	−0.69	0.21	2.2-13.6
Flowers per node						
wet 2007, n = 64	1	83.1	18.49	−0.26	0.16	29.9-86.2
dry 2006, n = 41	9	47.1	17.52	−0.25	0.24	44.8-50.1
wet 2006, n = 48	12	48.3	13.76	−0.15	0.20	34.2-48.3
wet 2007, n = 64	19	9.6	13.83	−0.23	0.15	2.9-13.6
Fruit set^1^						
dry 2007, n = 41	8	50.2	20.01	0.33	0.29	32.4-57.4
Proportion of flowers that set fruit						
dry 2007, n = 39	11	0	12.17	0.17	0.16	0-9
dry 2007, n = 39	13	39.2	13.32	−0.18	0.16	30.8-59.2
wet 2006, n = 29	14	0	19.99	0.26	0.35	0-4.8

^1^measured as proportion of clones.

^2^measured as percentage sterile pollen.

Traits assessed in different environmental conditions (wet/dry) and in different years (2006/2007) are noted, along with the number of individuals analyzed for each trait (n). Effects in BCIB are the result of *I. fulva *alleles and effects in BCIF are the result of *I. brevicaulis *alleles. Location of each QTL is presented as the linkage group (LG) followed by position on the linkage group (in Kosambi cM). The likelihood ratio (LR), the additive effect, percentage of variance explained (R^2^) and the 2 LOD confidence interval are also presented.

## Discussion

Over the past 25 years, the Louisiana Irises have emerged as an ideal system in which to ask questions regarding speciation, hybridization, and adaptation [29]. Drawing on previous studies examining the evolution, ecology, and genetics of Irises [4,9,15,26,29], we mapped QTL for 11 traits important for pre- and post-zygotic isolation using two maps derived from reciprocal backcrosses based on co-dominant EST-SSR markers [30]. Given the high degree of collinearity of the two maps, we have been able to compare the location of QTL across both maps for the first time. Despite the presence of multiple reproductive isolating barriers, the Louisiana Iris genome appears tolerant to introgression at multiple loci [20,31] and many of the markers used in this study have shown evidence of transmission ratio distortion with a bias towards introgression of *I. fulva* alleles, while *I. brevicaulis* alleles are under represented [30].

Understanding the genetic architecture underlying traits important to reproductive isolation and hybrid fitness allows us to develop hypotheses regarding which genetic regions are important for maintaining species distinctions and which may provide a selective advantage when allowed to introgress through hybridization in nature. Here we discuss the genetic relationship among 11 traits that affect pre- and post-zygotic isolation between two closely related species, *I. brevicaulis* and *I. fulva* inferred by QTL mapped using collinear, reciprocal-backcross genetic maps.

### Floral phenology

Previous studies have shown that flowering time in natural populations of *I. fulva* and *I. brevicaulis* acts as a strong pre-zygotic isolating barrier, with only a small proportion of the latest flowering *I. fulva* overlapping with the earliest flowering *I. brevicaulis* for approximately 2 weeks in late-April and early May [19,21]. Across both maps, we found 13 QTL for flowering time. At all eight loci detected in the BCIB population that were associated with variation in flowering time, individuals with introgressed *I. fulva* alleles flowered earlier than those with the *I. brevicaulis* allele, and conversely, for all 5 flowering time QTL detected in the BCIF population, introgression of the *I. brevicaulis* alleles resulted in an increase in the time to flowering. Two pairs of these QTL overlap on homologous linkage groups 4 and 13 in both backcross maps (Figure1, Table 1). These overlapping QTL suggest the potential for allelic differences at the same locus affecting flowering time between *I. brevicaulis* and *I. fulva*, although the confidence intervals for each QTL are large, encompassing many genes, leaving the possibility that each overlapping QTL may actually represent different loci affecting flowering time. Within each backcross population, several QTL from different years or treatments overlapped, indicating that there may be loci responding to specific developmental factors (e.g. first vs. second year post transplant) or environmental cues (e.g. wet vs. dry). For example, QTL detected in the dry site in both 2006 and 2007 overlap in both mapping populations (LG 1 in the BCIF population and LG 4 in the BCIB population; Figure1), suggesting that these loci may function to control flowering time in dry conditions. Another pair of overlapping QTL on linkage group eight in the BCIB mapping population were identified using data collected from both the wet and the dry plots in 2006 that may represent either a common locus responding to particular environmental conditions experienced in 2006 or, perhaps, a locus that controls variation in flowering during the first year.

Genetic studies in model systems have shown that flowering time is a complex trait responding to both endogenous and environmental cues, with loci that promote and delay flowering interacting to establish proper timing [32]. The 2006 phenological data using a dominant *Iris* retroelement (IRRE) marker system was previously analyzed [4] and although most of the QTL that were identified in that study had effect directions consistent with what is found in this study (i.e. *I. brevicaulis* alleles cause later floral transition, *I. fulva* alleles cause earlier floral transition), several QTL with opposite effect directions (e.g. *I. brevicaulis* alleles that cause earlier floral transition, *I. fulva* alleles that cause later floral transition) were identified [4] that we were unable to detect in this study. This may be attributed to the fact that their study had slightly larger sample sizes and identified a greater overall number of flowering time QTL [4].

### Flood tolerance and long-term survival

The habitat commonly associated with Louisiana Irises fluctuates dramatically both throughout the year, and year-to-year, as water levels and temperatures fluctuate. Under changing conditions, it is expected that plants will have environment dependent responses that may appear under stressful conditions (e.g. flood, drought). We evaluated data for both long-term survival in mildly fluctuating conditions as well as survival in extreme flooding conditions in the backcross mapping populations using data from a transplanted field plot that experienced standard environmental fluctuations after 3 years and another plot that experienced abnormally strong flooding. Only two survival QTL were detected: one QTL associated with variation in flood tolerance and one QTL associated with long-term survival. As would be predicted from the habitat associations of the two species [20,33], introgression of the *I. brevicaulis* allele into the *I. fulva* genetic background at either of these loci resulted in decreased survivorship. Two QTL linked to increased survival in the BCIF mapping population that were identified in a previous study were not recovered here [9]. As in the previous studies that analyzed both survival in the greenhouse and the flood survival data using dominant markers, no loci affecting survival were identified in the BCIB populations in this study [9].

### Sterility

The BCIB mapping population exhibits higher pollen sterility (32.3 % mean sterility; range 0.58 – 100 % sterile) relative to the BCIF population (7.56 % mean sterility; range 0.18-66.7 % sterile). These BCIB values contrast with the parents used to generate the crosses as Ib 25, If 174, and the F1 hybrid all had pollen sterility less than 10 %. We detected two QTL in the BCIB mapping population in which introgression of the heterospecific (*I. fulva*) allele resulted in a decrease in the proportion of sterile pollen grains and one QTL in the BCIF mapping population in which introgression of the heterospecific (*I. brevicaulis*) allele resulted in an increase in pollen sterility. The location of the QTL on BCIB LG 3 associated with a decrease in sterility also corresponds to a region with significant transmission ratio distortion (TRD) whereby *I. fulva* alleles were overrepresented in the BCIB mapping population [30], consistent with heterozygosity being favored in this region. The other two QTL associated with sterility do not correspond with regions exhibiting TRD. A potential explanation for the increase in fertility in the BCIB individuals associated with the introgression of either of the two *I. fulva* loci is that homozygosity in *I. brevicaulis* may have negative effects (i.e. due to inbreeding depression). Both iris species demonstrate a mixed-mating reproductive strategy, but *I. brevicaulis* demonstrates lower levels of inbreeding than does *I. fulva*[21,34]. This pattern of mating would be predicted to result in more heterozygosity in *I. brevicaulis*, and indeed this species does demonstrate higher levels of heterozygosity relative to *I. fulva*[30,35]. Given that a proportion of the heteroyzgosity in these species involves deleterious recessive alleles, we would predict that our crossing design would uncover more deleterious alleles in *I. brevicaulis* than in *I. fulva*. Specifically, both backcross populations would have higher levels of homozygosity than would be present in the progeny of natural outcrossing individuals, potentially revealing loci that cause sterility when homozygous [36]. Alternatively, the fact that each backcross population was created from a different F_1_ individual (F_1_2 and F_1_3) may contribute to differential levels of sterility in the backcross populations. Interspecific incompatibilities between the species could also explain the increase in sterility in the BCIB mapping population, although evidence supporting either negative interactions between heterospecific nuclear genes or cytonuclear incompatibilities has not been found [30]. Further crossing experiments among and within *I. fulva* and *I. brevicaulis* individuals should help to elucidate the potential causes for pollen sterility.

### Growth traits affecting fitness

It is well documented that *I. brevicaulis* and *I. fulva* occur in different habitats, indicating that they vary in physiological attributes [9,15,19,33]. In addition, they also differ in vegetative and floral traits that may affect fitness [26,27,35]. The interplay between genetic pathways controlling physiology and architecture are likely important for controlling variation in these traits [37]. For example, the ability to generate carbon and nutrient stores may affect the ability of plants to produce more branching points, but this trait is also controlled by genes important for determining the location and frequency of branch production [38]. While developmental pathways controlling branching (number of growth points produced, number of nodes per inflorescence), the transition to flowering (inflorescence present/absent), and fruit production all have unique downstream genetic components, these pathways are also dependent to some extent on physiological processes such as resource accumulation [38-41]. Therefore, it would be expected that some QTL for these traits would be independent and specific to the downstream pathway involved, but some affecting physiology may be shared.

Relative to the previous analysis using dominant markers (i.e. [26]), far fewer QTL were detected in this study (~1/3 as many for BCIB and ~1/2 as many for BCIF). Interestingly, in very few instances were overlapping QTL for the same growth trait identified across study year and habitat (BCIB LG 4, BCIF LG 5, LG 11), however in several cases, overlapping QTL for different growth traits were identified. The limited number of overlapping QTL detected in this study could potentially result from the independent genetic architecture underlying these traits. Alternatively, the small sample size for some of these traits limits our power to detect QTL, so QTL of small effect that may actually be overlapping are not detected. As such, the degree of overlap reported herein is likely a conservative estimate of QTL overlap.

In the plants examined for this study, the number of growth points produced before the flowering season increased in both backcross populations from 2006 to 2007 as the newly planted rhizomes became more established [26]. Although the increased number of growth points translated into a higher likelihood of flowering, this increase in the number of new growth points actually resulted in a lower proportion of growth points that produced an inflorescence [26]. This phenomenon may be explained by the co-occurrence of QTL on BCIF LG 8 where introgression of *I. brevicaulis* alleles results in a decrease in the number of growth points produced by weight and an increase in the proportion of growth points that produce an inflorescence (Figure1).

QTL for two traits, whether or not an inflorescence is formed (flowering) and the number of nodes produced per inflorescence (branching), are found in the same regions across several individual linkage groups. This can be seen on linkage groups BCIB LG 11 and BCIF LG 3. In both cases, the heterospecific allele is associated with decreases in the trait values. On LG 11, two QTL detected in the BCIB population are in a homologous position with a QTL detected in the BCIF population associated with variation in the number of floral nodes per inflorescence, indicating that *I. brevicaulis* has an allele in this region that increases inflorescence and floral node production in both genetic backgrounds. A similar pattern is seen on the end of LG 2 where QTL were detected in which the *I. fulva* allele is associated with increased inflorescence production (in the BCIB population) and higher numbers of floral nodes per inflorescence (in the BCIF population). It is possible that a few genetic regions controlling resource acquisition explain QTL for both of these traits.

One interesting pattern is that all of the QTL in this study associated with the number of flowers per node have negative additive effects, even those identified in both populations on homologous regions of LGs 1 and 19. Usually a second flower is produced at a node only after flowers at all other nodes have fully developed [42]. Therefore, the trait ‘number of flowers per node’ is likely to be affected by whether or not there is enough energy to produce flowers at all nodes and then begin to produce secondary flowers at nodes. In parental populations, *I. fulva* produces more flowers per node [26] and we do not have an explanation for why all of the QTL identified in this study, independent of cross direction, have negative additive effects unless heterozygosity at each of these loci results in decreased trait values.

### Overall genomic architecture of pre-zygotic isolation and hybrid fitness

Introgression of traits in a hybrid zone is dependent on the genetic architecture underlying traits affecting isolation [43,44]. If QTL underlying traits that contribute to isolation are dispersed throughout the genome, a greater proportion of the genome will be linked to these loci, decreasing the potential for the introgression of beneficial QTL while maintaining species boundaries. However, when these factors are clustered, the likelihood of introgression across the genome is increased, especially if there are positive fitness effects of the donor allele on the recurrent parent [14]. Additionally, introgression is influenced by the effect size and direction of alleles at clustered loci. Most of the QTL identified in this study are relatively evenly distributed across the genome. In this study, flowering time and sterility are the primary traits affecting isolation. The flowering time QTL are distributed throughout the genome, but introgression of any of the alleles identified in this study would have the effect of decreasing reproductive isolation between the parental taxa, weakening this key species isolation barrier. The effect of this shift on fitness is complicated and likely differs depending on other genetic and environmental variables. On BCIB LG 4, QTL for four different traits are located in a relatively small region. These traits include flowering time, sterility, and fruit set – all of which have the potential to affect reproductive isolation and fitness. In this region, introgression of the heterospecific allele increases the fitness of the introgressed individual in that it increases the number of nodes per inflorescence and fruit production while decreasing sterility, suggesting that this region may likely introgress across species boundaries in nature, although this introgression may have the added effect of decreasing isolation through flowering time. One region of the genome that would likely experience selection against introgression is on LG 9 where *I. brevicaulis* alleles in the *I. fulva* background increase sterility and are linked to additional QTL decreasing fitness (fewer inflorescences per growth point and fewer flowers per node). However, QTL increasing sterility are not widespread in this study, suggesting that this trait only restricts introgression in a small portion of the genome.

## Conclusions

Several patterns emerge from using the linked backcross maps to identify QTL associated with pre-zygotic isolation, survivorship, and fitness in crosses between *I. fulva* and *I. brevicaulis*. Overall, we observe that there are fewer QTL than were found for many of the same traits in previous studies, however, this is quite likely due to the smaller sample sizes associated with the EST-SSR markers. Despite this, we observe some trends including the aggregation of QTL in some regions suggesting that the QTL affecting fitness/reproductive isolation have similarities in their genetic architecture across the *I. fulva* and *I. brevicaulis* genomes. Moreover, we hypothesize that the clustering of QTL on certain linkage groups is likely to ‘protect’ traits important for fitness from being separated by recombination. Finally, this study draws on cross year, treatment, and environment data, which allows us to better understand how traits interact with each other, as well as with the environment. Future studies will be able to use the rich framework of these linked QTL maps to better understand adaptive introgression in Louisiana Irises. These loci serve as hypotheses for patterns of introgression in nature, which are currently being addressed.

## Methods

### Construction of mapping populations and linkage maps

Reciprocal first generation backcross (BC_1_) mapping populations were generated between wild-collected individuals of *I. brevicaulis* and *I. fulva*. The parental *I. brevicaulis* genotype (Ib25), used as the maternal parent, was collected from an oak hardwood forest in St. Martin Parish, Louisiana, while the parental *I. fulva* genotype (If174), used as the paternal parent, was collected along bayou margins in Terrebonne Parish, Louisiana. The clonal nature of these irises allowed for multiple clones of genetically identical plants to be used to generate the backcross populations. Multiple clones of two F_1_ individuals (F_1_3 and F_1_2) were used as the pollen parents and crossed to clones of Ib25 and If174, respectively, to generate first generation backcross populations to *I. brevicaulis* (the ‘BCIB’ population) and *I. fulva* (the ‘BCIF’ population). Seeds from these crosses were planted in 1999 and were repotted from a single rhizome each subsequent year.

A subset of the backcross individuals, 94 BCIB and 92 BCIF, were used to generate linked high-density genetic linkage maps using 232 and 237 co-dominant EST-SSR markers, respectively [30]. For this study, the remaining backcross plants were genotyped at a subset of the EST-SSR markers. 239 BCIB plants were genotyped for 131 markers and 168 BCIF plants were genotyped for 123 markers (118 shared markers) distributed across each map. Genotyping of the microsatellite markers was also as described in [30]. New maps were generated from these data using Mapmaker 3.0 [45-47]. Initial framework maps for each backcross population were generated using a likelihood odds (LOD) threshold of 7.0 and a maximum recombination frequency threshold of 0.4. Decreasing the LOD threshold to 5.0 and 3.0 allowed for the remaining unlinked markers to be incorporated into the maps. Map distances (cM) were calculated using the Kosambi mapping function. The maps utilized in the current study have thus been modified slightly from those published in [30].

### Assaying trait values

The following traits were examined in this study: (1) flowering time, (2) flood tolerance, (3) long-term survival, (4) sterility, (5) number of growth points (branches on the rhizome) produced before the flowering season, (6) presence/absence of an inflorescence on each plant, (7) proportion of growth points that produced inflorescences, (8) number of flowering nodes produced per inflorescence, (9) number of flowers produced per node, (10) whether or not flowering plants set fruit, and (11) proportion of flowers that set fruit. The data for most of these traits (2, 5–11) have been previously reported and QTL for these traits were mapped on non-linked genetic maps created with a dominant marker system. Some data for flowering phenology, hybrid sterility and long-term survival are presented for the first time here.

Traits 1–3, and 5–11 were all assayed in field conditions that relied on similar experimental plots located in southern Louisiana. Generally, these experimental plots were set up in a similar manner in which several ramets from the same individual of every BCIB and BCIF plant were transplanted into evenly spaced (0.5 m) randomized positions. Differences between these previous experimental plots, highlighted below, involve the specific location of each plot, the exact number of clones and genotypes present in each plot, and the years and conditions in which various traits were assayed.

Flood tolerance was assayed in an experimental field site along the edge of the Choupique Bayou in the Atchafalaya Basin Floodway in Louisiana (as described in [9]). In 2005, this field site experienced flooding of an abnormally long duration that acted as an extremely strong selective agent. Multiple clonal replicates for each of 185 BCIB genotypes and 209 BCIF genotypes, totaling 416 BCIB and 357 BCIF individuals were assayed for flood tolerance, defined as the proportion of clonal replicates that survived this flooding event.

Flowering phenology and growth traits affecting fitness (traits 5–11 above) were assayed in two field plots also located along the Choupique Bayou in the Atchafalaya Basin Floodway (the same plots described in [4,23,26]. These two field plots were qualified as “wet” and “dry,” with the “wet” plot remaining inundated with shallow water for a much longer duration after heavy rains. From 1–5 clones for each of 243 BCIB and 172 BCIF genotypes were planted into each plot in 2005. In 2006 and 2007, flowering phenology and the growth traits affecting fitness were measured in each plot. Flowering phenology for each plant was measured in days after the date at which the first flower was observed each year (data for 2006 have been analyzed previously in [4], data from 2007 have not been previously analyzed). The number of new growth points was counted prior to the flowering season each year (January 2006 and March 2007) and we controlled for the original weight of the planted rhizome. Plants were scored for whether or not they produced an inflorescence, the proportion of ramets that produced inflorescences, the number of flowering nodes produced per inflorescence, the number of flowers produced per node, whether or not each flowering plant set fruit, and the proportion of flowers per plant that set fruit, as described in [26]. Long-term survival was assessed in another nearby plot, located along the Choupique Bayou in the Atchafalaya Basin Floodway. A total of 1200 backcross hybrid plants (average 2.4 clones/genotype) were transplanted into this plot in October 2008 and examined for survival in the spring of 2011.

Pollen sterility was assessed in 194 BCIF and 258 BCIB genotypes in the spring of 2001. Pollen grains were collected from one anther per plant. The proportion of fertile pollen grains was assessed by examining pollen stained with a solution of lactophenol-aniline blue [48] and counting the number of both fertile and infertile pollen grains. Lactophenol-analine blue stains starch molecules of potentially viable pollen grains, while inviable pollen grains with no starch do not uptake the stain [48]. Approximately 500 pollen grains per plant were scored to estimate levels of sterility among the parental and backcross genotypes.

### QTL analysis

For each trait, when data were available for multiple clones per genotype, trait values were averaged across clones in a per plot and per annum basis (i.e. trait values for all clones per genotype in the wet plot in 2006 were averaged). Averaging across clonal replicates renders nominal traits, such as flood tolerance, long-term survival, and the presence/absence of inflorescences and fruits, as continuous characters. The distribution of a quantitative trait is likely determined by several underlying QTL, each of which can affect the phenotype in a number of different ways. Therefore, applying transformations to normalize data from this “mixture distribution” is not appropriate for QTL mapping studies, and such transformations were not applied to data in this study [49,50]. Windows QTL Cartographer version 2.5 [51] was used to conduct composite interval mapping (CIM; [52]) on the data to identify QTL. CIM was carried out using forward and backward regression at 2-cM intervals separately on the BCIB and BCIF maps. A 10-cM window was used to exclude closely linked cofactors, and the number of control markers was set at five (the default program setting). For each trait, 300 permutations were run to calculate the genome-wide significance threshold for declaring a QTL. The locations of significant QTL are reported as the map location of the point where the LR statistic is the greatest, including confidence intervals of 2-LOD on either side. When two QTL peaks for the same trait occurred in close proximity on the same linkage group, a drop below the permutation cut-off or a change in the directionality of the QTL effect was used to determine whether each peak represented different QTL. The directionality of the effects (whether or not heterospecific alleles increased or decreased trait values), additive effect sizes, and proportion of variance explained (R^2^) of QTL were also calculated using CIM.

Genotypic information for use in the QTL analyses was only available for plants that survived until 2009 when they were genotyped for the linkage maps. Consequently, the sample sizes in the current analyses are somewhat smaller than sample sizes in previous analyses. Calculations of the percent variation explained by a given QTL are known to be over-estimated when sample sizes are small [53], therefore, we are more interested in interpreting the directionality of the QTL and the relationship among the QTL for the traits of interest. Values for both directionality and the percentage of variance (R^2^) explained for each trait are given in Table 2.

## Authors’ contributions

ST and SJK performed genotyping. ESB and ST generated the linkage map. SJT and NHM collected phenotypic data from the field. MLA, AB, and ANB collected sterility data. ESB, ANB, SJT, and NHM performed QTL analyses. MLA and NM designed and coordinated the study. ESB and ANB wrote the manuscript with feedback from NHM, SJT, and MLA. All authors read and approved the final manuscript.
